# Update to: Study Pre-protocol for “BronchStart - The Impact of the COVID-19 Pandemic on the Timing, Age and Severity of Respiratory Syncytial Virus (RSV) Emergency Presentations; a Multi-Centre Prospective Observational Cohort Study”

**DOI:** 10.12688/wellcomeopenres.16778.4

**Published:** 2024-12-18

**Authors:** Thomas C. Williams, Steve Cunningham, Simon B. Drysdale, Helen Groves, Dalia Iskander, Xinxue Liu, Mark D. Lyttle, Robin Marlow, Abigail Maxwell-Hodkinson, Chengetai D. Mpamhanga, Shaun O'Hagan, Ian Sinha, Olivia V. Swann, Thomas Waterfield, Damian Roland, Paediatric Emergency Research in the UK and Ireland (PERUKI)

**Affiliations:** 1 Institute of Genetics and Cancer, University of Edinburgh, Edinburgh, UK; 2 Centre for Inflammation Research, University of Edinburgh, Edinburgh, UK; 3 Department of Paediatric Respiratory and Sleep Medicine, Royal Hospital for Children and Young People, Edinburgh, UK; 4 Oxford Vaccine Group, Department of Paediatrics, University of Oxford, Oxford, UK; 5 NIHR Oxford Biomedical Research Centre, Oxford, UK; 6 Wellcome-Wolfson Institute for Experimental Medicine at Queen’s University Belfast, Belfast, UK; 7 Department of Anthropology, University College London, London, UK; 8 Faculty of Health and Applied Sciences, University of the West of England, Bristol, UK; 9 Emergency Department, Bristol Royal Hospital for Children, Bristol, UK; 10 Bristol Royal Hospital for Children, Bristol, UK; 11 University of Liverpool Medical School, Liverpool, UK; 12 Department of Child Life and Health, University of Edinburgh, Edinburgh, UK; 13 University of Liverpool, Liverpool, UK; 14 Alder Hey Children's Hospital, Liverpool, UK; 15 Department of Paediatric Infectious Diseases and Immunology, Royal Hospital for Children, Glasgow, UK; 16 Paediatric Emergency Medicine Leicester Academic (PEMLA) Group, Leicester Royal Infirmary, Leicester, UK; 17 Sapphire Group, Health Sciences, Leicester University, University of Leicester, UK

**Keywords:** COVID-19, Respiratory Syncytial Virus, Bronchiolitis, Infants, Children, Palivizumab

## Abstract

**Background:**

In 2021 we launched the BronchStart study, which collected information on 17,899 presentations in children with serious respiratory tract infections following the release of lockdown restrictions. Our study informed the Joint Committee on Vaccination and Immunisation’s decision to recommend the introduction maternal respiratory syncytial virus (RSV) vaccination, which was introduced in the United Kingdom in August/September 2024.

**Study question:**

We modified our original protocol to conduct a United Kingdom-wide assessment of maternal vaccination against RSV.

**Methods and likely impact:**

We will conduct a multi-centre study, utilising the PERUKI network used in the original BronchStart study, to assess the effectiveness of maternal vaccination using a test-negative study design. We will gather detailed clinical information on children admitted with bronchiolitis in the post-RSV vaccination era, and understand possible reasons for incomplete vaccine uptake.

## Introduction

The BronchStart study ^1– 3^ was launched in 2021, anticipating an increase in serious respiratory infections in children following the release of lockdown measures implemented to limit the spread of the Covid-19 pandemic. Since then it has documented serious early life respiratory disease in the United Kingdom for the past three years, collecting detailed information bronchiolitis admissions, and feeding into the Joint Committee for Vaccination and Immunisation (JCVI) decision to introduce widespread RSV immunisation to the UK ^4^. Maternal RSV vaccination has now been recommended across the United Kingdom, and was rolled out from August 12 ^th^ 2024 (Scotland ^5^) and September 1 ^st^ 2024 (England ^6^, Wales ^7^, Northern Ireland ^8^) for pregnant women who are at a gestation of 28 weeks or more.

Once vaccination is introduced, understanding vaccine effectiveness (VE) will be a key clinical and policy priority. National approaches to assessing VE will be conducted by the public health agencies of the Four UK Nations (England, Scotland, Wales and Northern Ireland). However, these are likely to be complicated by data linkage methodological issues that will delay analyses and release of data to well after the end of the winter RSV season; equally these analyses will be unable to collect detailed clinical information on demographics and outcomes for those who have/have not been the recipients of maternal vaccination. In addition, they will not be able to understand barriers to uptake of maternal RSV vaccination.

The aim of this update to the BronchStart protocol is to leverage the research infrastructure created for BronchStart to evaluate effectiveness of RSV maternal vaccination in a real-world setting during the first season (2024–2025) of maternal RSV introduction in the UK. To achieve this the BronchStart protocol has been amended to include a test-negative design (TND) VE study. Below we list key modifications to the original protocol required to conduct a test-negative VE study, with an embedded survey of mothers to understand potential barriers to vaccine uptake.

## Study protocol

The original study protocol ^1^ was structured in keeping with the principles of the STROBE statement (strobe-statement.org/). This update to the protocol details any changes made to this original protocol for a study of maternal RSV vaccine effectiveness (VE) in the 2024/2025 winter season.

### Sample selection

A national multi-centre prospective observational cohort study will be carried out under the auspices of the PERUKI (Paediatric Emergency Research in the UK and Ireland) Network ^9^. All children meeting the inclusion criteria below will be eligible for the vaccine effectiveness study, which will be nested within the original study.

***Inclusion criteria***: The maternal RSV vaccine has been made available to all pregnant mothers from August 12 ^th^ 2024 (Scotland) and September 1 ^st^ 2024 (England, Wales, Northern Ireland). Therefore eligible participants will be infants born after the August 12 ^th^ 2024 (Scotland) or September 1st 2024 (England, Wales, Northern Ireland) admitted to a hospital with clinical features of bronchiolitis (cough, tachypnoea or chest recession, and wheeze or crackles on chest auscultation) ^10^, lower respiratory tract infection (clinical diagnosis) or a first episode of acute viral wheeze (for the detailed rationale for only recruiting infants/children presenting with their first episode of wheeze, please refer to V2 of the protocol).

***Exclusion criteria***: Children with previous episodes of wheeze responsive to bronchodilator, suggesting an underlying diagnosis of recurrent wheeze of early childhood; this is likely to represent a much smaller patient group than the original BronchStart study, due to a focus in this updated protocol on infants born after the 12 ^th^ August 2024 (Scotland) or 1 ^st^ September 2024 (England/Wales/Northern Ireland) entering their first RSV winter season (2024/5).

### Data collection

We will collect data at two time points: baseline (date of presentation to a participating hospital) and seven days later. Informed consent will be sought from mothers of eligible infants for permission to conduct a questionnaire, and access their medical records for their immunisation status. Clinicians identifying a case for inclusion will keep a local log of participants that contain patient identifiable characteristics cross referenced to a study number. An email after 7 days to the submitting clinician will prompt data entry at this point.

Data on the vaccine effectiveness sub-study will be entered to a secure online database (REDCap data capture tool) ^11, 12^ (see below).

### Variables to be measured

At baseline, data including patient demographics, presenting characteristics, acuity and results from point of care virology testing will be collected (see Supplementary File 1). An external link will enable clinicians to enter a full postcode derived index of multiple deprivation score for database entry.

At 7 days data will include the infant’s length of stay (if this is longer than 7 days, further reminder emails will be sent on a weekly basis) highest acuity dependency (the ward they were placed on if admitted: Observation Unit, Normal, High Dependency or Intensive Care), whether care the patient was discharged or died and (if obtained) what viruses were identified by PCR (see Supplementary File 1).

RSV status will be identified by nasopharyngeal aspirate/swab (NPA/NPS) tested by either (a) point of care testing (rapid viral testing where available) at baseline presentation to ED, or (b) by laboratory PCR testing, if either is performed as part of standard care.

To understand factors associated with maternal RSV vaccine uptake, we consulted with a medical anthropologist (DI) to design a questionnaire for mothers of infants participating in the study. The final questionnaire included questions that adhered to the principals of the Five 5C’s model ^13, 14^ of vaccine hesitancy, which posits that psychological antecedents such as confidence, complacency, constraints, calculation, and collective responsibility influence vaccine uptake on an individual level.

### Sample size calculation

Two approaches were taken for sample size calculations: one based on the WHO recommendations for the evaluation of Covid-19 vaccine effectiveness ^15^, and a second simulating a variety of different vaccine effectiveness and coverage rates in the populations recruited in our previous studies, bootstrapping our proposed analyses methods to determine the confidence intervals we would obtain of the ‘known’ vaccine effectiveness. 

***Calculations based on WHO recommendations for evaluation of VE.*** Calculations were based on the precision of the VE estimated by the test-negative design, as recommended by the WHO, and implemented using their VE calculator ^16^. The maternal vaccine coverage of Tdap is just under 60% in England ^17^ and maternal RSV vaccine coverage could be as low as 30% in the first season. Assuming the true VE for RSV-associated bronchiolitis/LRTI hospitalisation among infants from birth through 6 months of age is 70%, the study would need to recruit 145 RSV-associated hospitalisations, with 1:1 matching with test-negative controls to achieve a precision width of 40% (+/-20%) for the VE.

***Calculations based on varying assumptions.*** Using an alternative method (epiR:epi.sscc) ^18^ it can be seen ( Table 1) that the number of cases required is very dependent on these assumptions being correct; the exact number is lower than previously as a precision width for the VE is not specified. As VE or uptake rates decline, the number of cases required rapidly rise.

**Table 1.** How the number of cases required varies by different assumptions of vaccine effectiveness and uptake. Calculated using epiR::epi.sscc ^18^ with following parameters: OR = 1-VE, p0 = coverage, power = 0.9).

	Vaccine Effectiveness					
Uptake	40%	50%	60%	70%	80%	90%
**5%**	2,161	1,287	828	560	391	280
**10%**	1,114	659	420	281	195	137
**15%**	768	451	285	189	129	90
**20%**	597	348	218	143	97	66
**25%**	498	287	178	116	77	52
**30%**	433	248	153	98	64	43
**40%**	361	203	123	77	49	31
**50%**	329	182	108	66	40	24
**60%**	326	177	102	61	36	20

***Sample size feasibility and number of recruitment sites required.*** Reviewing case recruitment for infants aged 0–6 months for the 2021/2 BronchStart season showed that for the top 5 recruiting centres between 54–111 RSV positive cases, and 40–78 RSV negative cases, were recruited by site over the peak 6 months of the season. For the 2024/5 season, a limited eligible patient population (infants born after August 12 ^th^ 2024 [Scotland] and September 1 ^st^ 2024[England/Wales/Northern Ireland]) and a likely reduction in respiratory presentations with the introduction of vaccination means that multiple recruiting centres will be required to meet the proposed sample sizes. Assuming a worst-case scenario of 20% uptake and 60% effectiveness (218 cases, 218 controls, total 436 cases), and that each centre were able to recruit 50 patients (25 cases, 25 controls), we aimed to identify at least 10 recruiting centres (~500 recruits) for this study.

### Study registration

The study will be registered on ISCRTN Registry ( https://www.isrctn.com/).

### Outcomes

***Primary outcome***: Estimate vaccine effectiveness (VE) of RSV maternal vaccination during pregnancy against RSV-associated LRTD hospitalization among infants from birth through 6 months of age.

a. Cumulative from birth through 6 months
b. Stratified by from birth to 3 months and from >3 to 6 months

***Secondary outcomes***

a. Describe level of care among hospitalized cases and controls (observation unit, hospital ward, high-dependency unit, paediatric intensive care unit)
b. Describe respiratory support among hospitalized cases and controls (low-flow oxygen, high-flow oxygen therapy, continuous positive airway pressure, bilevel positive airway pressure, and invasive mechanical ventilation)
c. Describe treatment modalities and frequencies (antibiotics, intravenous fluid, nasogastric fluid)
d. Evaluate RSV testing rates and modality during the 2024–2025 RSV season in ED and hospital settings across the PERUKI network to inform feasibility of VE assessments against other outcomes in future RSV seasons using test negative designs.
e. Describe predictors of maternal vaccine uptake (including but not limited to prematurity, logistical challenges, maternal choice).

### Data analysis and statistical plan

Our primary analysis will be an un-matched conditional logistic regression stratified by site and calendar month of attendance with age, prematurity and sex as co-factors. Stratifying by site and month is important to control for local differences in vaccine uptake. We will also carry out a matched (by site, age and date of attendance) analysis as a secondary analysis, but anticipate that close chronological matching of cases and controls may prove not possible due to the sharp peaks in attendance, due initially RSV cases as the epidemic spikes, then due to non-RSV disease caused by other viruses as RSV recedes. We will look at vaccine effectiveness for all infants and also the subgroup of those whose mother received a vaccine >14 days before birth.

A thematic analysis will be conducted on free text responses collected in the maternal questionnaire. Data will be coded to identify patterns and themes, and a codebook developed comprising codes and sub-codes generated from etic and emic categories, descriptions of each, and examples of representative data. 

### Ethical issues

The original BronchStart study was a non-consented study which only used routinely collected clinical data on the infant with respiratory disease. However, as for this study we would be accessing maternal, as well as infant, health records, we felt that consultation with a parent and patient group was necessary to understand how to collect this information in a way that was acceptable to parents of children with serious respiratory disease.

Prior to designing the study, we therefore launched a consultation with a patient and public involvement (PPI) group for parents of children affected by viral wheeze, Fourteen parents responded, located in England and Northern Ireland. We asked whether, as a mother, respondents would be happy for researchers to check maternal medical records to verify their vaccination history. None of the respondents stated that they would not be happy for a researcher to access their medical records. Five out of 14 (36%) stated that they would be happy for researchers to access their medical records without needing to be told about it; 7/14 (50%) were happy for researchers to access their medical records with verbal consent; 2/14 (14%) were happy for researchers to access their medical records, as long as they were provided with written information and able to provide written consent. We therefore proceeded on the basis of providing appropriate information and eliciting consent for the VE sub-study. The study was submitted for Integrated Research Application System (IRAS) approval with University Hospitals of Leicester NHS Trust as the Study Sponsor, IRAS ID 297802, and received a favourable opinion from the Research Ethics Committee on the 8 ^th^ August 2024. The consent form for the study is available as Supplementary File 2, and the study information leaflet as Supplementary File 3.

### Data input, storage and management

Data will be entered using the validated online data entry software REDCap. (Research Electronic Data Capture tools) following the clinical report forms provided in the appendix (Supplementary Files 1). This software (REDCap) is hosted on the University Hospitals Bristol and Weston NHS Foundation Trust (UHBW) secure server, accessible on the Health and Social Care Network (HSCN) that is managed by NHS Digital. All research data reside within the hosting institution. The study Sponsor Organisation (University Hospitals of Leicester NHS Trust) will be the Data Controller throughout, and University Hospitals Bristol and Weston NHS Foundation Trust will have Joint Controllership for the duration of data entry and cleaning. REDCap uses a granular security model so that users can only review the data they have been explicitly authorised to access. REDCap also provides a comprehensive log/audit feature that records all individual changes with a date/time stamp and a change owner. Data that are captured and stored will only be available via the information technology systems linked to the HSCN which is the current validated system used by NHS Trusts to share and store patient information.

### Dissemination

Data will be presented in:

- A real-time study dashboard ( https://bronchstop.netlify.app/)
- Data submissions to the regulatory authorities, public health agencies and local study teams.
- A study preprint
- A peer reviewed scientific journal
- Engagement with the PPI group involved as part of study design, and the Resvinet Patient Network, who were involved in the original BronchStart study

## Conclusions

Within a rapid timeframe, we anticipated that this study will generate estimates of vaccine effectiveness which will help to inform planning for subsequent RSV seasons. In addition, detailed information will be collected on infant demographics, clinical presentations and outcomes in the post-RSV vaccination era. Additionally, insights into associations with incomplete maternal vaccine administration will facilitate future efforts to boost vaccine uptake.

## Data Availability

As this is a study protocol no data are yet available. Study data will be stored on a RedCap server hosted by University of West of England, Bristol, United Kingdom (See Data input, storage and management). Anonymised, aggregate data will be shared with interested parties upon reasonable request following approval from the sponsor institution (UHL NHS Trust). Data are available under the terms of the Creative Commons Attribution 4.0 International license (CC-BY 4.0). Edinburgh Research Archive: BronchStart Study Extended Data [ https://era.ed.ac.uk/handle/1842/37604?show=full] Available here will be: BronchStop_supplementary_file1.pdf (Consent form for BronchStop sub-study) BronchStop_supplementary_file2.pdf (Parent information leaflet for BronchStop sub-study) BronchStop_supplementary_file3.pdf (BronchStop sub-study CodeBook)
