# Supplementary material for: Update to: Study Pre-protocol for “BronchStart - The Impact of the COVID-19 Pandemic on the Timing, Age and Severity of Respiratory Syncytial Virus (RSV) Emergency Presentations; a Multi-Centre Prospective Observational Cohort Study”
Source: Wellcome Open Res. 2024 Dec 18;6:120. Originally published 2021 May 19. [Version 4] doi: 10.12688/wellcomeopenres.16778.4 (PMC8378404; doi:10.12688/wellcomeopenres.16778.4)
Supplement: Peer review history

# Reviewer report: Reviewer response for version 4

**DOI:** 10.21956/wellcomeopenres.25971.r115143

**Published:** 2025-01-18

**Authors:** Ram Hari Chapagain

**Affiliations:** 1 National Academy of Medical Sciences (NAMS), Kathmandu, Nepal

The  Introduction ,methodology along with inclusion and exclusion criteria  and variables need to be collected is clearly mentioned. the outcome variable is mentioned clearly. The statistical plan is  also clear. 

 It is better to have consent form parents  before collecting the data as consenting is major in any form of study.

Is the study design appropriate for the research question?

Yes

Is the rationale for, and objectives of, the study clearly described?

Yes

Are sufficient details of the methods provided to allow replication by others?

Yes

Are the datasets clearly presented in a useable and accessible format?

Not applicable

Reviewer Expertise:

Vaccine trials , Burden study ,  economic study

I confirm that I have read this submission and believe that I have an appropriate level of expertise to confirm that it is of an acceptable scientific standard.

# Reviewer report: Reviewer response for version 4

**DOI:** 10.21956/wellcomeopenres.25971.r116019

**Published:** 2024-12-27

**Authors:** Michael G Ison

**Affiliations:** 1 National Institute of Health, Rockville, MD, USA

The authors propose collecting a range of data that will inform maternal decision making about RSV vaccine (retrospectively), rates of testing for RSV and VE from this observational cohort.  Similar RWE studies have been helpful in understanding the clinical utility of these new vaccines.  Some issues that were unclear to me:

 1.  How well does the cohort reflect the UK population that it is targeting to assess?

 2.  There are differences in sensitivity of antigen-based vs. PCR-based testing.  It is unclear how that will be factored in as a negative antigen test could miss a case.

 3.  Likewise, involvement requires consent - what is the expected consent rate and will the consented patients likely reflect the overall population or be skewed?

 4.  Is there variability of level and type of care across hospitals - this could result in skewed data if there is heavier enrollment in some centers.

Is the study design appropriate for the research question?

Yes

Is the rationale for, and objectives of, the study clearly described?

Yes

Are sufficient details of the methods provided to allow replication by others?

Yes

Are the datasets clearly presented in a useable and accessible format?

Yes

Reviewer Expertise:

Expert in RSV vaccines, respiratory viruses in adults and immunocompromised hosts.

I confirm that I have read this submission and believe that I have an appropriate level of expertise to confirm that it is of an acceptable scientific standard.

# Reviewer report: Reviewer response for version 4

**DOI:** 10.21956/wellcomeopenres.25971.r115941

**Published:** 2024-12-23

**Authors:** Susan Hopkins

**Affiliations:** 1 UK Health Security Agency, London, UK

The updates to this article address my major concerns.

Is the study design appropriate for the research question?

Partly

Is the rationale for, and objectives of, the study clearly described?

Yes

Are sufficient details of the methods provided to allow replication by others?

Yes

Are the datasets clearly presented in a useable and accessible format?

Partly

Reviewer Expertise:

Infectious Diseases, Epidemiology, Vaccine Effectiveness studies.

I confirm that I have read this submission and believe that I have an appropriate level of expertise to confirm that it is of an acceptable scientific standard.

# Reviewer report: Reviewer response for version 3

**DOI:** 10.21956/wellcomeopenres.25532.r107417

**Published:** 2024-11-13

**Authors:** Susan Hopkins

**Affiliations:** 1 UK Health Security Agency, London, UK

This is an important study which aims to assess vaccine effectiveness of maternal RSV vaccine to prevent serious RSV illness (by reviewing hospitalisations) in babies from 0-6 months). It also aims to provide some assessment of level of care required, respiratory support, associated treatment, evaluate testing and maternal factors in uptake of vaccination.

 The introduction states that national datasets will be unable to assess vaccine effectiveness. I am unsure how this statement is made as UKHSA provides vaccine effectiveness estimates for influenza, COVID-19 and would be expected to do that for RSV. 

 The additional qualitative overlay in this study is beneficial and would not be performed by routine national studies. 

 Given this PERUKI group is UK and Ireland, I would observe it was an opportunity to compare the different policy approaches to vaccination - maternal vaccination in the UK and neonatal vaccination in Ireland - it would enable an assessment of the different approaches in similar health systems and provide a natural experiment.

 The precision width of 40% (+/- 20%) is very wide. Why is it only possible to recruit 10 sites? Improving the precision estimate would be much better.

 The number of cases and controls recruited is small and therefore the ability to determine the impact of vaccination at various points in pregnancy, lack of ability to provide chronological matching and improved matching of cases and controls will be a limiting factor.

 I did not have access to the data variables detected and therefore could not assess the suitability of the data collected. There was no assessment on how well the data collection planned has been completed in previous studies.

 Overall, this study will add value particularly on describing the detail of healthcare delivery, the testing approaches within hospitals and the qualitative work on maternal vaccination. However, it will only provide a limited assessment on VE.

Is the study design appropriate for the research question?

Partly

Is the rationale for, and objectives of, the study clearly described?

Yes

Are sufficient details of the methods provided to allow replication by others?

Yes

Are the datasets clearly presented in a useable and accessible format?

Partly

Reviewer Expertise:

Infectious Diseases, Epidemiology, Vaccine Effectiveness studies.

I confirm that I have read this submission and believe that I have an appropriate level of expertise to confirm that it is of an acceptable scientific standard, however I have significant reservations, as outlined above.

# Response

**Published:** 2024-12-16

**Authors:** Thomas Williams

We did not mean to imply in our introduction that public health agencies such as UKHSA or Public Health Scotland would not be conducting studies to evaluate vaccine effectiveness. We have therefore edited the wording of the Introduction to make this clearer (additions underlined): “Once vaccination is introduced, understanding vaccine effectiveness (VE) will be a key clinical and policy priority. National approaches to assessing VE will likely be conducted by the public health agencies of the Four UK Nations (England, Scotland, Wales and Northern Ireland). However, these are likely to be complicated by data linkage methodological issues that will delay analyses and release of data to well after the end of the winter RSV season.” Initially we had hoped to compare, using the same methodology, outcomes in both the UK and the Republic of Ireland with the introduction of maternal RSV vaccination/nirsevimab, but unfortunately issues related to funding and the timing of ethical approvals made this impractical. We have applied for funding to perform this comparison, if this is successful the protocol will be amended for next season. The accuracy of the VE in our study was based on the worst-case scenario that maternal RSV vaccine coverage is 30%. This means that, in the worst-case scenario, the study can provide a VE with a lower confidence interval >50% assuming the true VE is 70%, i.e. we will have 95% confidence to conclude the VE is >50% based on the above assumptions. The lower boundary of VE in pivotal vaccine trials is normally between 0% - 30%. Therefore, we felt the precision of VE in our study is acceptable. If the vaccine coverage is as high as the Tdap coverage, the precision of VE will be +/- 15%. The rapidly evolving nature of the study meant that we could only guarantee that a limited number of sites would be able to come on board in time to start recruiting for the 2024/5 RSV season. However, although we aimed for a minimum of 10 sites, we recognised that the larger the number of sites, the higher the prevision of estimates, and we are pleased to report that we currently have over 20 sites recruiting to the study ( https://bronchstop.netlify.app/ )

 We recognise that the small planned sample size is likely to limit our ability to conduct sub-analyses of study data.

 We apologise for the lack of access to the data variables: there was a delay in uploading the data variables sheet to the University of Edinburgh repository. This is now available at this link: http://dx.doi.org/10.7488/era/885 We agree that the most comprehensive assessment of VE will come from a large national study of routinely collected data on hospital admissions. However we hope that our study will offer additional value over and above the information that can be established from such a national study.

# Reviewer report: Reviewer response for version 3

**DOI:** 10.21956/wellcomeopenres.25532.r105179

**Published:** 2024-10-29

**Authors:** Sarah Geoghegan

**Affiliations:** 1 Paediatric Infectious Diseases, Children's Health Ireland (Ringgold ID: 575376), Dublin, Leinster, Ireland; 2 University College Dublin, Dublin, Leinster, Ireland

In this protocol  the PERUKI research group present an important and timely update to the BronchStart study. With this update, the group will aim to evaluate vaccine effectiveness of maternal RSV vaccine against RSV associated LRTD hospitalization in infants from birth through 6 months and also stratified from birth to 3 months and 3-6 months. This is a timely and important study, particularly given the UK will be among one of the only countries this season to implement a maternal vaccine strategy alone. Data on vaccine effectiveness of the RSV maternal vaccine as well as determinants of maternal vaccine uptake are much needed to help inform RSV immunization policy.

 The authors should be commended for their efforts to make their study protocol and data collection tools available and to rapidly disseminate data via a real-time study dashboard. 

**Suggestion:**

 The Inclusion and exclusion criteria are outlined, however the rationale for exclusion of children with previous episodes of wheeze responsive to bronchodilator is not described, I suggest the authors include their rationale for this decision in the published protocol.

Is the study design appropriate for the research question?

Yes

Is the rationale for, and objectives of, the study clearly described?

Yes

Are sufficient details of the methods provided to allow replication by others?

Yes

Are the datasets clearly presented in a useable and accessible format?

Not applicable

Reviewer Expertise:

Vaccine implementation. Clinical Epidemiology. Vaccine uptake.

I confirm that I have read this submission and believe that I have an appropriate level of expertise to confirm that it is of an acceptable scientific standard.

# Response

**Published:** 2024-12-16

**Authors:** Thomas Williams

We thank our reviewer Dr. Geoghegan for their positive response to the updated protocol. In the updated version of the protocol we have added a link to the study dashboard for this winter season: https://bronchstop.netlify.app/**

 In the initial BronchStart study, we were interested to see whether a change in the age at which infants/children experienced their first RSV infection was associated with a change in phenotype. Due to the lockdown measures implemented to prevent the spread of respiratory viruses, we speculated that many children would experience their first RSV infection between the ages of 1 and 2  years, rather than, as in a normal season, between the age of 0-1. We speculated that this might manifest as a wheezing phenotype rather than typical bronchiolitis. However, we did not want to capture repeat attendances for the children who present with frequent wheeze episodes, so limited this group by specifying that we only wanted to capture the first wheezing episode (which could be a manifestation of first RSV infection). For this updated version to the protocol, the oldest child likely to be recruited to the proposed vaccine effectiveness study is around 6 months of age, as we will only capture children born from the 12 ^th^ August onwards who present at any point up until the end of the RSV season, which tends to come to a close in February/March. Therefore we felt that this was likely to be less of a concern than in earlier iterations of the study, but we have maintained this wording to ensure consistency with previous versions of the protocol. Please see the changes to the wording below, with additions underlined. “Inclusion criteria : The maternal RSV vaccine has been made available to all pregnant mothers from August 12 th 2024 (Scotland) and September 1 st 2024 (England, Wales, Northern Ireland). Therefore eligible participants will be infants born after the August 12 th 2024 (Scotland) or September 1st 2024 (England, Wales, Northern Ireland) admitted to a hospital with clinical features of bronchiolitis (cough, tachypnoea or chest recession, and wheeze or crackles on chest auscultation) 10 , lower respiratory tract infection (clinical diagnosis) or a first episode of acute viral wheeze (for the detailed rationale for only recruiting infants/children presenting with their first episode of wheeze, please refer to V2 of the protocol). Exclusion criteria : Children with previous episodes of wheeze responsive to bronchodilator, suggesting an underlying diagnosis of recurrent wheeze of early childhood; this is likely to represent a much smaller patient group than the original BronchStart study, due to a focus in this updated protocol on infants born after the 12th August 2024 (Scotland) or 1st September 2024 (England/Wales/Northern Ireland) entering their first RSV winter season (2024/5).”

# Reviewer report: Reviewer response for version 1

**DOI:** 10.21956/wellcomeopenres.18503.r44077

**Published:** 2021-08-19

**Authors:** Patrick K. Munywoki

**Affiliations:** 1 Centers for Disease Control and Prevention, Nairobi, Kenya

With a primary objective to assess the impact of COVID-19 on RSV transmission and disease severity, this surveillance study in the UK is topical and important. The protocol is well designed and the methods are fully described. I have just minor comments.

1. Introduction: Reference(s) for statements on long-term effects RSV is missing. Kindly add.
2. Data analysis: Please confirm if you will use multivariate analysis or if it will be multivariable logistic regression with binary outcomes of hospitalization, level of ventilatory support and PICU admission examined separately. Currently it is not clear. 

Is the study design appropriate for the research question?

Yes

Is the rationale for, and objectives of, the study clearly described?

Yes

Are sufficient details of the methods provided to allow replication by others?

Yes

Are the datasets clearly presented in a useable and accessible format?

Not applicable

Reviewer Expertise:

RSV; Infectious disease epidemiology

I confirm that I have read this submission and believe that I have an appropriate level of expertise to confirm that it is of an acceptable scientific standard.

# Reviewer report: Reviewer response for version 1

**DOI:** 10.21956/wellcomeopenres.18503.r44084

**Published:** 2021-06-23

**Authors:** Simon Drysdale

**Affiliations:** 1 St George's University Hospitals NHS Foundation Trust, London, UK

This is a protocol for a timely study in the UK. The study is well planned and pragmatic for busy EDs. I have minor comments only:

- Collection of data on participants’ attendance at childcare / nursery etc would be useful.
- For the 7 day follow up, I assume families are not being contacted again but researchers are just looking at their local hospital record. Will you know if they attend at a different hospital (or GP) in the follow up period? This may need to be noted as a limitation.
- For the stratification of severity did you consider also using a scoring system (e.g. the ReSViNet score - I think you are collecting all the required data anyway?).
- It is unclear to me how "excess morbidity" will be defined / measured.
- On the supp file 2: units for H+ (pH) listed as mmHg, is this correct? (not nmol/L on nM)?

Is the study design appropriate for the research question?

Yes

Is the rationale for, and objectives of, the study clearly described?

Yes

Are sufficient details of the methods provided to allow replication by others?

Yes

Are the datasets clearly presented in a useable and accessible format?

Not applicable

Reviewer Expertise:

RSV, respiratory viruses, paediatrics

I confirm that I have read this submission and believe that I have an appropriate level of expertise to confirm that it is of an acceptable scientific standard.
